# LINC01287/miR-298/STAT3 feedback loop regulates growth and the epithelial-to-mesenchymal transition phenotype in hepatocellular carcinoma cells

**DOI:** 10.1186/s13046-018-0831-2

**Published:** 2018-07-13

**Authors:** Yichao Mo, Longguang He, Zeru Lai, Zhiheng Wan, Qinshou Chen, Sibo Pan, Liangfu Li, Dasheng Li, Junwei Huang, Fan Xue, Siyao Che

**Affiliations:** 1 Department of Hepatobiliary Surgery, Gaozhou People’s Hospital, Gaozhou, China; 2 Department of General Surgery, The First Affiliated Hospital of BaoTou Medical University, Baotou, Inner Mongolia China

**Keywords:** LINC01287, miR-298, STAT3, Hepatocellular carcinoma

## Abstract

**Background:**

The long non-coding RNAs (lncRNAs) have participated in the promotion of hepatocellular carcinoma (HCC) initiation and progression. Nevertheless, the biological role and underlying mechanism of LINC01287 in HCC has never been reported.

**Methods:**

The TGCA database was used to explore the abnormal expression of lncRNAs in HCC. Real-time PCR and in situ hybridization assays were used to examine the expression of LINC01287 in HCC tissues. The clinicopathological characteristics of HCC patients in relation to LINC01287 expression were then analyzed. Infection of cells with the si-LINC01287 lentiviral vector was performed to down-regulate LINC01287 expression in HCC cells. MTT and colony formation assays were performed to examine cell growth ability, and FACS analysis was performed to examine the cell cycle and apoptosis. A Boyden assay was used to examine HCC cell invasion ability, and RNA immunoprecipitation tested the interaction between LINC01287 and miR-298. A luciferase reporter assay was used to examine whether STAT3 was a direct target of miR-298, and chromatin immunoprecipitation (ChIP) was used to examine the potential binding of c-jun to the miR-298 promoter.

**Results:**

We revealed that the expression of LINC01287 was increased in HCC cell lines, as well as tissues. Knockdown of LINC01287 decreased HCC cell growth and invasion both in vitro and in vivo. LINC01287 can negatively regulate miR-298 expression by acting as a ceRNA. miR-298 directly targeted STAT3 and inhibited its expression. LINC01287 exerted its function via the miR-298/STAT3 axis in HCC. Interestingly, STAT3 elevated LINC01287 expression via c-jun, which bound to the LINC01287 promoter. A feedback loop was also discovered between LINC01287 and the miR-298/STAT3 axis.

**Conclusions:**

Our data indicated that LINC01287 played an oncogenic role in HCC growth and metastasis and that this lncRNA might serve as a novel molecular target for the treatment of HCC.

**Electronic supplementary material:**

The online version of this article (10.1186/s13046-018-0831-2) contains supplementary material, which is available to authorized users.

## Background

Ranking the fifth most common cancer, Hepatocellular carcinoma (HCC) is the third leading cause of cancer-related death worldwide [1]. Lots of HCC patients are at the late stages of the disease when they are diagnosed. In addition, there is a high frequency of tumor recurrence in HCC patients although they have surgical resection and this contributes to the poor prognosis of HCC [2]. Therefore, it is urgent in need to develop novel strategies for the diagnosis and treatment of HCC.

Approximately 2% of the human genome accounts for protein coding genes. However, the majority of transcripts consists of non-coding RNAs (ncRNAs) [3] that can be grouped into the following classes depending on their transcript size: long non-coding RNAs (lncRNAs) and small ncRNAs [4]. LncRNAs are transcripts with a length greater than 200 nucleotides (nt). The abnormal expression of lncRNAs often contributes to tumor initiation, growth and metastasis [5]. Many studies have demonstrated that lncRNAs are dysregulated in many cancers, including HCC [6]. For instance, lncRNA CRNDE is upregulated in HCC and significantly associated with poor clinical outcomes, and knockdown of its expression impairs cell proliferation and invasion [7]. In addition, lncRNA TUG1 contributes to HCC cells proliferation, migration and tumorigenesis via interacting with miR-144 [8]. Moreover, NEAT1 upregulates TGF-β1 to induce HCC progression by sponging hsa-miR-139-5p [9]. Although large numbers of lncRNAs have been annotated, the role and molecular regulatory mechanisms of lncRNAs in HCC still require further clarification.

MicroRNAs (miRNAs), a group of small and non-coding RNAs, regulate down-stream targets expression via modulation of post-transcriptional [10].Mounting evidence has demonstrated that miRNAs regulate diverse biological process, including cancer cell proliferation, apoptosis and invasion [11]. It is worth to note that lncRNAs can function as ceRNAs that compete for miRNA binding, and therefore, they derepress the expression of miRNA-targeted mRNAs [12]. The lncRNA-miRNA-mRNA regulatory network has been implicated to regulate tumorigenesis [13, 14].

In the current study, we explore the role and underlying mechanism of LINC01287 in HCC. We also explore the interaction between LINC01287 and miR-298.

## Methods

### Cell culture and collection of HCC patient samples

HCC cell lines (HepG-2, Huh7, Bel7402 and Hep3B) and the normal liver epithelial cell line LO2 were purchased from the Institute of Biochemistry and Cell Biology of the Chinese Academy of Sciences (Shanghai, China). All cell lines were maintained at 37 °C in a humidified 5% CO_2_ atmosphere in RPMI-1640 medium supplemented with 10% fetal bovine serum.

HCC tissues and matched normal tissue samples (98 pairs, formalin-fixed and paraffin-embedded) were obtained from patients at Gaozhou People’s Hospital. These patients were diagnosed with hepatocellular carcinoma in our hospital from Sep. 2011 to Nov. 2016. Written informed consent was obtained from all patients, and the project was approved by the Ethical and Scientific Committees of Gaozhou People’s Hospital. All experimental protocols were approved by the Clinical Research Ethics Committees of Gaozhou People’s Hospital.

### Cell transfection, lentivirus production and transduction

The cell transfections were performed using lipofectamine 2000 reagent (Invitrogen) according to the manufacturer’s instructions. siRNA against STAT3 (Ruibio, Guangzhou, China) and a non-targeting siRNA control (Ruibio, Guangzhou, China) were used to knock down gene expression. Briefly, the oligonucleotides and plasmids were transfected using Lipofectamine 2000 (Invitrogen) according the manufacturer’s protocol. 48 h later, G418 was added into the medium to select of stable clones. The pcDNA3.1-STAT3 and pcDNA3.1 vectors were purchased from Santa Cruz Biotechnology (USA). miR-298, miR-ctrl, anti-miR-ctrl and anti-miR-298 were purchased from Genechem (Shanghai, China).

The shRNA sequence that targeted LINC01287 was AAGCATTGTAGACCTGGCTGCTGAA. The sequences were cloned into the pGFP-C-shLenti vector according to the manufacturer’s instructions (Origene). Then, the viruses were packaged in 293 T cells according to a standard protocol. HCC cells were infected with virus particles plus 6 μg/ml Polybrene.

### Quantitative real-time PCR (q-RT-PCR)

Total RNA was extracted from HCC cells using TRIzol reagent (Invitrogen) according to the manufacturer’s instructions. The RNA was reverse transcribed to cDNA, which was followed by real-time PCR analyses. The primers used in the study are listed in Table 1.

**Table 1:** Primers used in the study

	Forward:5’-3’	Reward:3’-5’
GAPDH	TCAAGATCATCAGCAATGCC	CGATACCAAAGTTGTCATGGA
U6	ATACAGAGAAAGTTAGCACGG	GGAATGCTTCAAAGAGTTGTG
LINC01287	GGTTGATGTAAGGACCTCGT	GAGACCTTGTTTCATGTGTCG
MIR-298	TCAGGTCTTCAGCAGAAGC	TAGTTCCTCACAGTCAAGGA
STAT3	CGCACTTTAGATTCATTGATGC	AGGTGAGGGACTCAAACTG
LINC01287 P1	CGAGTACTTCTAAATCCCAGT	AAAGGGTTCTCTCACTAAAAG
LINC01287 P2	AGAAATCTATATTGACAGT	CCTGGGTAGGAATTGTAAGCGA
LINC01287 P3	ATAGGCTGAAATGCACTGAAC	GACAAAAACCGCAGCGAGCGG
LINC01287 P4	TCCGTGTGTGGTGGTACTGG	GATTAAAACATAAAAATCAT

To determine the sub-cellular distribution of LINC01287, nuclear and cytoplasmic fractions of cells were separated using the PARIS Kit (Life Technologies) according to the manufacturer’s instructions. Then, the RNA was extracted from both fractions. Subsequently, RT-PCR was performed to examine the expression ratios of specific RNA molecules between the nuclear and cytoplasmic fractions.

### MTT, colony formation and cell cycle assays

For the MTT assay, HCC cells were seeded in 96-well plates. After 24 h, 5 mg/ml MTT was added to each well. The HCC cells were then incubated for another 6 h. Subsequently, DMSO was added to each well, and the absorbance was measured at 490 nm by spectrophotometry.

For the colony formation assay, HCC cells were seeded in 6-well culture plates. Two weeks later, the cells were fixed in paraformaldehyde, stained with hematoxylin solution and counted under a microscope.

For the cell cycle assay, cells were harvested from the culture dishes and washed three times in cold PBS. The cells were then fixed in 70% ice-cold ethanol at 4 °C overnight. Finally, the cells were incubated in propidium iodide (supplemented with RNase A). A FACScaliber flow cytometry system (BD Biosciences) was used to examine the DNA content of labeled cells.

### Boyden assay

For the Boyden assay, HCC cells in serum-free DMEM were seeded in the upper chamber, which was inserted into a 24-well plate. RPMI-1640 supplemented with 10% FBS was added to each well. After 24 h, the HCC cells that remained on the upper surface of the membrane were removed, while the cells that migrated to the lower membrane were fixed in paraformaldehyde, stained with crystal violet and counted.

### Western blot and immunofluorescence (IF) assays

In the Western blot assay, the protein samples were transferred onto a PVDF membrane, which was followed by an incubation overnight at 4 °C in a 1:500 dilution of primary antibodies. Subsequently, the membrane was incubated with HRP-conjugated rabbit or mouse secondary antibodies for 1 h at room temperature and were then developed using a chemiluminescence reagent.

For the IF assay, cells were plated on culture slides. After 24 h, when the cells had adhered to the slides, the cells were rinsed in phosphate-buffered saline (PBS) three times and were then fixed in ice-cold methanol-acetone for 10 min. Subsequently, the cells were blocked for 10 min in 5% BSA in PBS, followed by incubation with the primary antibodies in PBS for 1.5 h at room temperature. After three washes in PBS, the slides were incubated with the secondary antibodies for 40 min. After three additional washes, the slides were stained with 4-, 6-diamidino-2-phenylindole (DAPI) for 10 min and were examined using an Olympus confocal imaging system.

### In situ hybridization assays

The in situ detection of lncRNA was performed as previously described [15].

### Chromatin immunoprecipitation (ChIP) assay

We performed the ChIP assay using a ChIP assay kit (Millipore, catalog: 17–371) according to the manufacturer’s instructions. Briefly, the cells were fixed with 1% formaldehyde to covalently crosslink the proteins to DNA, after which the chromatin was harvested from the cells. Subsequently, the crosslinked DNA was sheared by sonication (sheared to 200–1000 base pairs in length) and was subjected to an immunoselection process. Then, PCR was performed to measure the enrichment of DNA fragments of the putative c-JUN-binding sites within the LINC01287 promoter.

### Luciferase reporter assay

We cloned the full-length STAT3 cDNA (lacking the 3-UTR) into the eukaryotic expression vector pcDNA3.1 (Invitrogen). Subsequently, the 3’-UTR of STAT3 was amplified and cloned downstream of the firefly luciferase gene in the pGL3 vector (Promega); this vector was termed the wild type (WT) STAT3–3’-UTR. Using the GeneTailor™ Site-Directed Mutagenesis System (Invitrogen), we established site-directed mutagenesis of the miR-298 binding sites in the STAT3 3’-UTR. This vector was termed the mutant type (MUT) STAT3–3’-UTR. Subsequently, we co-transfected the HCC cells with the wt or mut STAT3–3’-UTR vector and the miR-298 mimic or inhibitor. Finally, we performed a luciferase assay using a dual Luciferase reporter assay system (Promega) 36 h after transfection. To perform LINC01287 promoter luciferase assays, HCC cells were seeded into 24-well plates and were co-transfected with plasmids that contained the LINC01287 promoter, the pRL-TK-Renilla plasmid (Promega, USA) and pcDNA.3-c-jun.

### In vivo tumor growth and invasion assay

All procedures involving animals were approved by the Institutional Committee on Animal Care of Gaozhou People’s Hospital. For tumor growth study, five mice were included into each group. Sh-ctrl, sh-LINC01287 or sh-LINC01287/anti-miR-298 cells were injected subcutaneously into both flanks of nude mice. Four weeks after implantation, the xenografts were removed from the mice and weighed. The tumor volume was calculated according to the following formula: 4π/3 × (width/2)^2^ × (length/2). The invasion assay was performed as previously described [16]. After 3 weeks, nude mice were evaluated for lung colonization capacity.

### Statistical analysis

SPSS 13.0 and Graph Pad Prism 5.0 software were used for the statistical analysis. The values are shown as the mean ± the standard error of the mean (S.E.M). Analyses of different groups were performed using one-way ANOVA or two-tailed Student’s t-test. *P* < 0.05 was considered statistically significant.

## Results

### LINC01287 expression was elevated in HCC tissues and cell lines

With the use of the online software program circlncRNAnet [17], we identified several lncRNAs that were significantly elevated in HCC tissues. Among these lncRNAs, LINC01287 was the most significantly different (Additional file 1: Figure S1A). Thus, we focused on LINC01287 for further analyses.

**Fig. 1:**
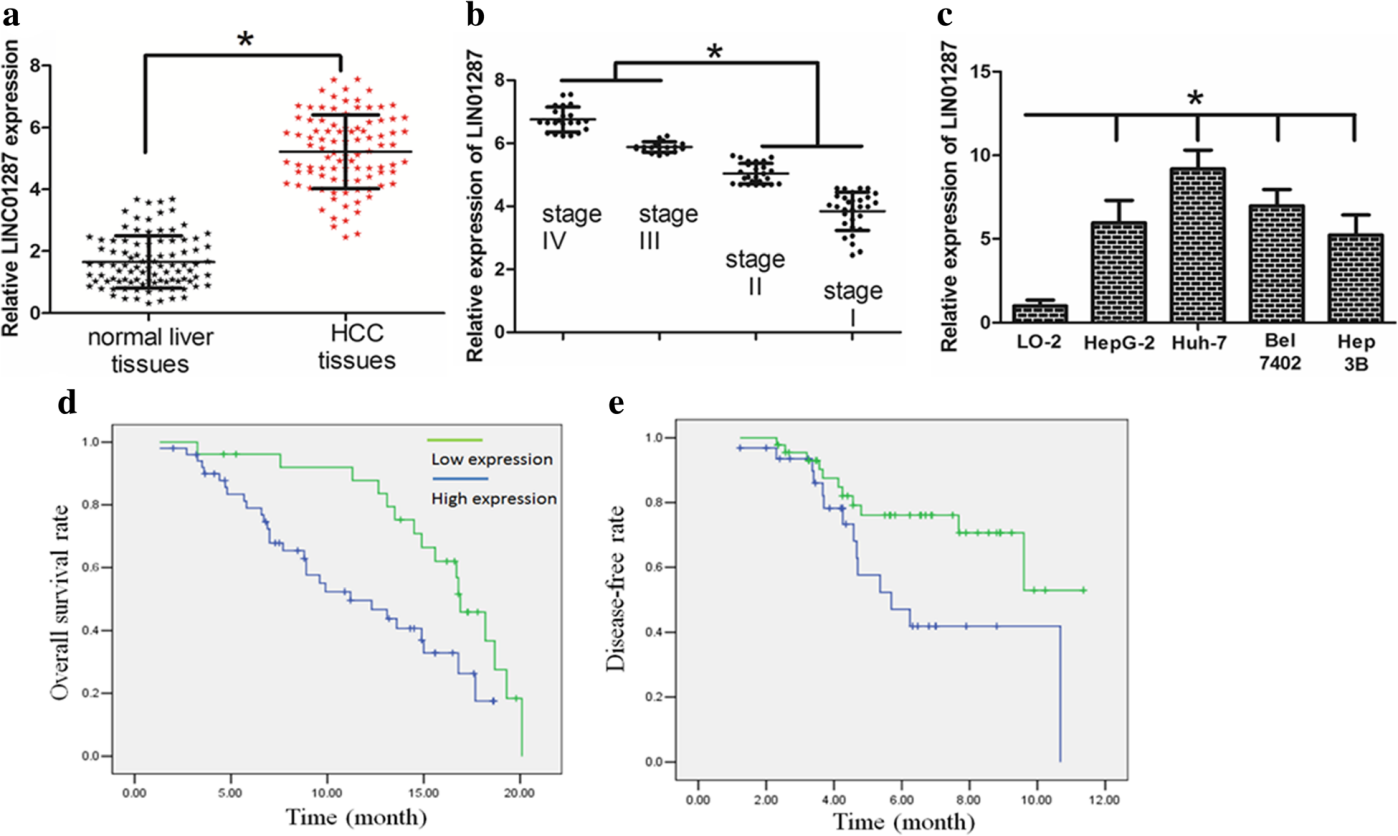
The LINC01287 expression level was up-regulated in HCC cell lines and tissues. **a** LINC01287 expression level was significantly up-regulated in primary HCC tissues. **b** The expression level of LINC01287 was up-regulated in patients with advanced stage HCC. **c** LINC01287 expression was higher in HCC cells compared with a normal liver cell line. **d** and **e** High expression of LINC01287 was associated with a shorter overall survival and a shorter disease-free survival time of HCC patients (blue and green curves represent low and high expression of LINC01287, respectively). * represents *P* value < 0.05

We further analyzed the expression level of LINC01287 in a larger cohort of 117 HCC patients. We revealed that the expression level of LINC01287 was up-regulated in HCC tissues compared with adjacent tissues (Fig. 1a). Interestingly, the expression level of LINC01287 was up-regulated in advanced-stage HCC (Fig. 1b) in comparison with early-stage HCC. In parallel, the expression level of LINC01287 was up-regulated in HCC cell lines compared with the normal human liver cell line LO2 (Fig. 1c).

We then questioned whether LINC01287 is indeed a non-coding RNA. An online bioinformatics analysis revealed that LINC01287 has no coding capability (http://cpc.cbi.pku.edu.cn/programs/run_cpc.jsp). The in vitro translation assay confirmed this finding (Additional file 1: Figure S1B). Subcellular fractionation and real-time PCR analysis were then performed to show that LINC01287 was primarily located in the cytoplasm (Additional file 1: Figure S1C).

### High expression of LINC01287 was associated with unfavorable prognosis

The association between LINC01287 expression and the clinicopathological features of the patients is shown in Table 2. The median value of LINC01287 expression was selected as the cut-off value for the separation of HCC patients with a low level of expression (49/98, 50%) from patients with a high level of expression of LINC01287 (49/98, 50%). Although LINC01287 expression was not associated with parameters such as age (*P* = 0.311), gender (*P* = 0.285) or HBV infection (*P* = 0.223), high LINC01287 expression was significantly correlated with tumor size (*P* = 0.001), lymph node metastasis (*P* = 0.026) and late clinical stage (*P* = 0.026). Interestingly, a Kaplan-Meier analysis found that a high expression level of LINC01287 was correlated with a shorter overall survival time and disease-free survival time in patients with HCC (Fig. 1d and e, *P* < 0.05).

**Table 2:** Associations between lncRNA LINC01287 expression and patients’ clinicopathological features

Variable	No. of patients	LINC01287 low expression	LINC01287 high expression	*P* value
Age				
< 60	45	20	25	0.311
≧60	53	29	24	
Gender				
Male	65	30	35	0.285
Female	33	19	14	
Tumor size				
< 5 cm	60	38	22	0.001
≧5 cm	38	11	27	
Lymph node involvement				
Absent (pN0)	53	32	21	0.026
Present (pN+)	45	17	28	
TNM stage				
I-II	47	29	18	0.026
III-IV	51	20	31	
HBV infection				
Yes	54	30	24	0.223
NO	44	19	25	

### Inhibition of LINC01287 decreased HCC cell proliferation and invasion

**Fig. 2:**
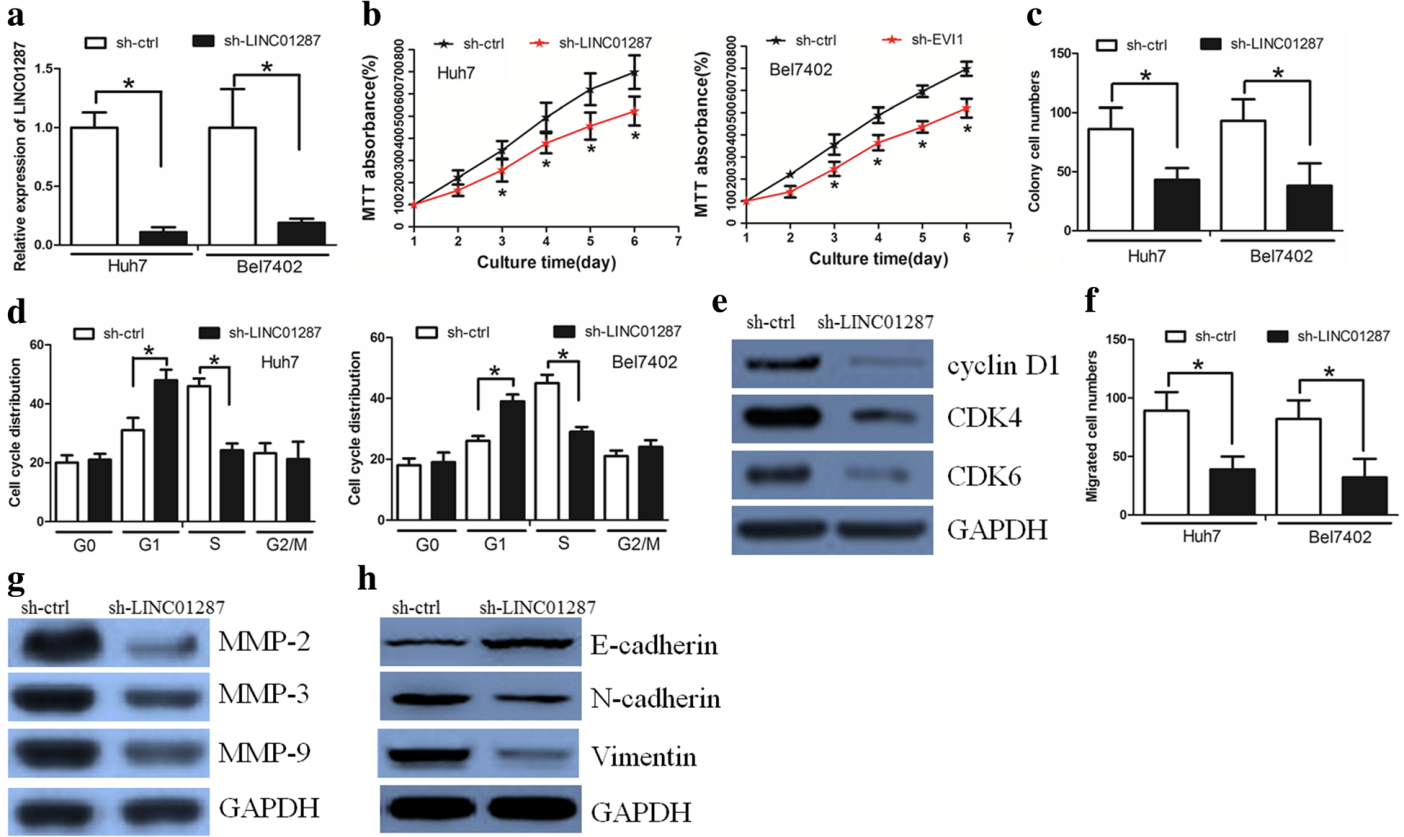
LINC01287 inhibition decreased HCC cell proliferation and invasion. **a** LINC01287 expression in Huh-7 and Bel7402 cells transduced with the control shRNA vector (sh-ctrl) or the LINC01287 shRNA vector (sh-LINC01287). **b** The MTT assay revealed that LINC01287 down-regulation significantly decreased cell proliferation. **c** The colony formation assay demonstrated that oncogenic survival was significantly decreased in sh-LINC01287 cells compared with sh-ctrl cells. **d** A higher frequency of sh-LINC01287 cells were in G1 phase and a lower frequency of these cells were in S phase. **e** LINC01287 down-regulation affected the expression of G1/S phase checkpoint proteins. **f** LINC01287 down-regulation decreased the invasion ability of HCC cells, as revealed by the Boyden assay. **g** The expression levels of MMP-2, MMP-3 and MMP-9 proteins were lower in sh-LINC01287 cells. **h** The expression level of E-cadherin was increased, while the expression levels of N-cadherin and Vimentin were decreased in sh-LINC01287 cells

Since Huh7 and Bel7402 cells exhibited the highest expression of LINC01287, we selected them for further studies. To investigate the biological function of LINC01287 in HCC cells, we established Huh7 and Bel7402 cells in which LINC01287 was stably knocked down (sh-LINC01287) (Fig. 2a). It was revealed that LINC01287 inhibition significantly decreased cell proliferation, as determined by MTT assay (Fig. 2b). By colony formation assay, we found that oncogenic survival was significantly inhibited in sh-LINC01287 cells compared with sh-ctrl cells (Fig. 2c, Additional file 2: Figure S2A). Flow cytometry analysis showed that sh-LINC01287 cells presented a significantly higher frequency of cells in G1 phase and a lower frequency of cells in S phase (Fig. 2d, Additional file 2: Figure S2B). Interestingly, the Western blot assay revealed that the expression levels of G1/S phase checkpoint proteins such as cyclin D1, CDK4 and CDK6 were significantly decreased in sh-LINC01287 cells (Fig. 2e).

Subsequently, we asked whether LINC01287 affected HCC cell invasiveness. The Boyden assay found that inhibition of LINC01287 decreased HCC cell invasiveness (Fig. 2f Additional file 2: Figure S2C). Consistently, according to the Western blot results, the expression levels of protein markers of invasion, including MMP-2, MMP-3 and MMP-9, were markedly decreased in sh-LINC01287 cells (Fig. 2g). Interestingly, the expression of epithelial to mesenchymal transition-related markers was also altered when LINC01287 was inhibited; E-cadherin expression was elevated, while N-cadherin and Vimentin expression was downregulated in sh-LINC01287 cells (Fig. 2h).

Overall, these data suggested that LINC01287 promotes HCC cell proliferation and invasion in vitro.

### LncRNA LINC01287 functioned as a ceRNA to regulate miR-298 expression

**Fig. 3:**
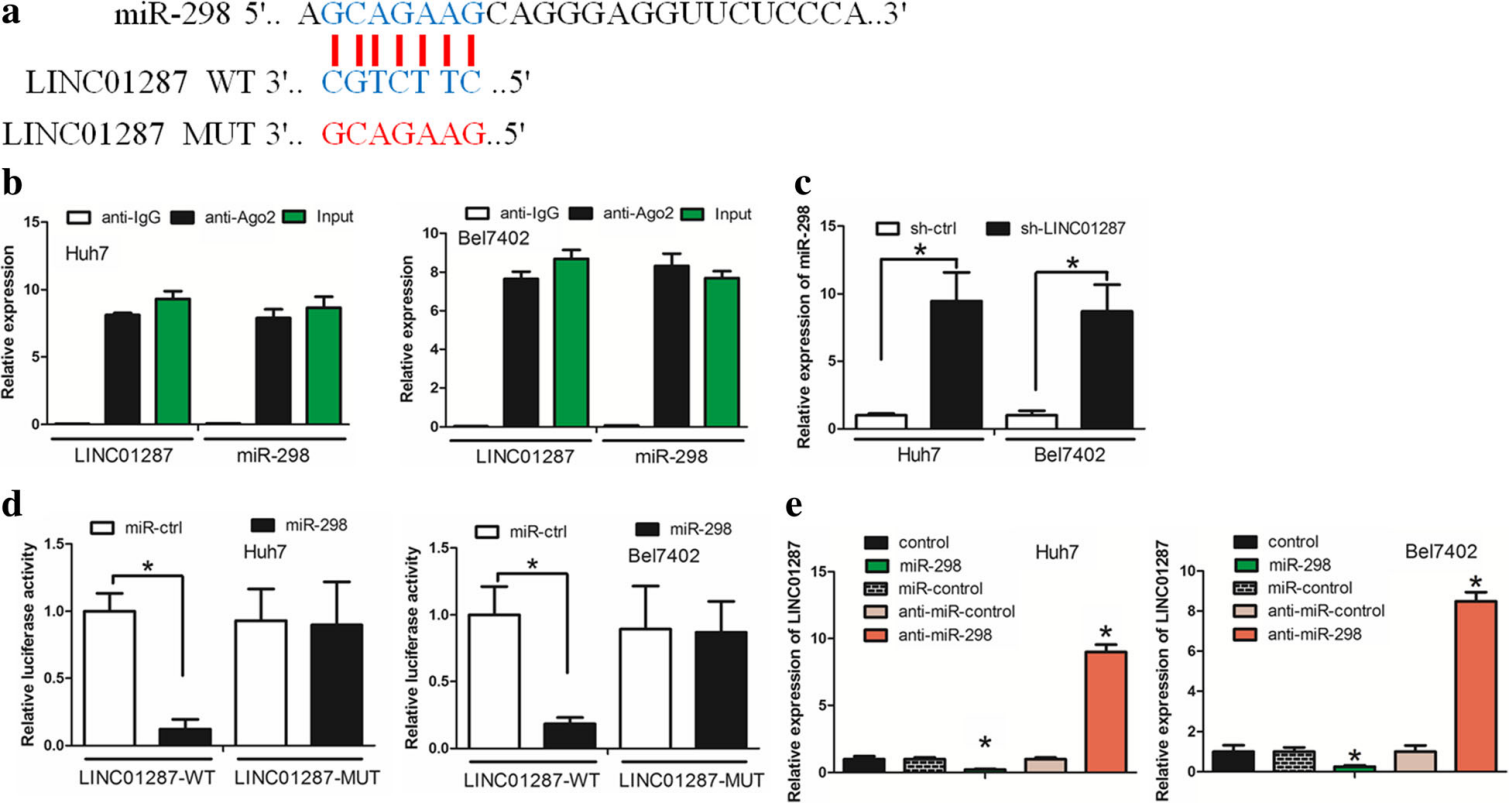
LncRNA LINC01287 acted as a ceRNA to regulate miR-298 expression. **a** The binding sites of miR-298 on LINC01287. **b** The RIP assay revealed that LINC01287 and miR-298 were enriched in the same Ago2 immunoprecipitates. **c** MiR-298 expression was increased in sh-LINC01287 cells compared with sh-ctrl cells. **d** Co-transfection of miR-298 and LINC01287-Wt strongly decreased the luciferase activity, while co-transfection of miR-ctrl and LINC01287-Wt did not change the luciferase activity. Co-transfection of miR-298 and LINC01287-Mut also did not change the luciferase activity. **e** MiR-298 decreased LINC01287 expression, while anti-miR-298 increased LINC01287 expression

LncRNA can exert its function by acting as a molecular sponge of miRNA [18]. We thus hypothesized that LINC01287 interacts with miRNAs in HCC. Using the online software miRDB (http://mirdb.org/miRDB/index.html), we searched for miRNAs with base pairs that are complementary to the LINC01287 sequence. We identified several microRNAs that may be regulated by LINC01287 (Additional file 2: Figure S2K). We first tested the difference in the expression of these microRNAs between the sh-ctrl and sh-LINC01287 groups of Huh7 cells. We found that only miR-298, miR-4308 and miR-23c were significantly altered between the sh-ctrl and sh-LINC01287 groups (Additional file 2: Figure S2L). In the sh-LINC01287 group, the level of miR-298 expression was increased approximately ten-fold higher compared with the sh-ctrl group, and consequently, we chose miR-298 for further study. The binding sites of miR-298 on LINC01287 are indicated in Fig. 3a. MiRNAs exert their gene silencing functions through a ribonucleoprotein complex, which is termed the RNA-induced silencing complex (RISC), the core component of which is Ago2 [19]. A radioimmunoprecipitation (RIP) assay was performed to examine whether LINC01287 and miR-298 are in the same RISC complex. It was revealed that LINC01287 and miR-298 were enriched in Ago2 immunoprecipitates compared with control IgG immunoprecipitates (Fig. 3b, *P* < 0.05). We then confirmed that when LINC01287 was down-regulated, the expression level of miR-298 was elevated in Huh7 and Bel7402 cells (Fig. 3c, *P* < 0.05). These data suggested that LINC01287 may negatively regulate miR-298. Subsequently, we examined whether miR-298 inhibited LINC01287 expression. The predicted miR-298 binding site of LINC01287 (LINC01287-WT) and a mutated miR-298 binding site of LINC01287 (LINC01287-MUT) were cloned into a reporter plasmid. We then performed luciferase assay to reveale whether miR-298 bound at LINC01287 and regulated LINC01287 expression. Co-transfection of miR-298 and LINC01287 decreased the luciferase activity, while co-transfection of miR-ctrl and LINC01287-WT and co-transfection of miR-298 and LINC01287-MUT did not change the luciferase activity (Fig. 3d, *P* < 0.05). In addition, we examined whether miR-298 regulated LINC01287 expression. It was revealed that, compared with the miR-ctrl, miR-298 inhibited LINC01287 expression. However, anti-miR-298 increased LINC01287 expression compared with anti-miR-ctrl (Fig. 3e, *P* < 0.05). Taken together, our data suggested a reciprocal interaction between LINC01287 and miR-298.

### LINC01287 regulated STAT3 expression through miR-298

**Fig. 4:**
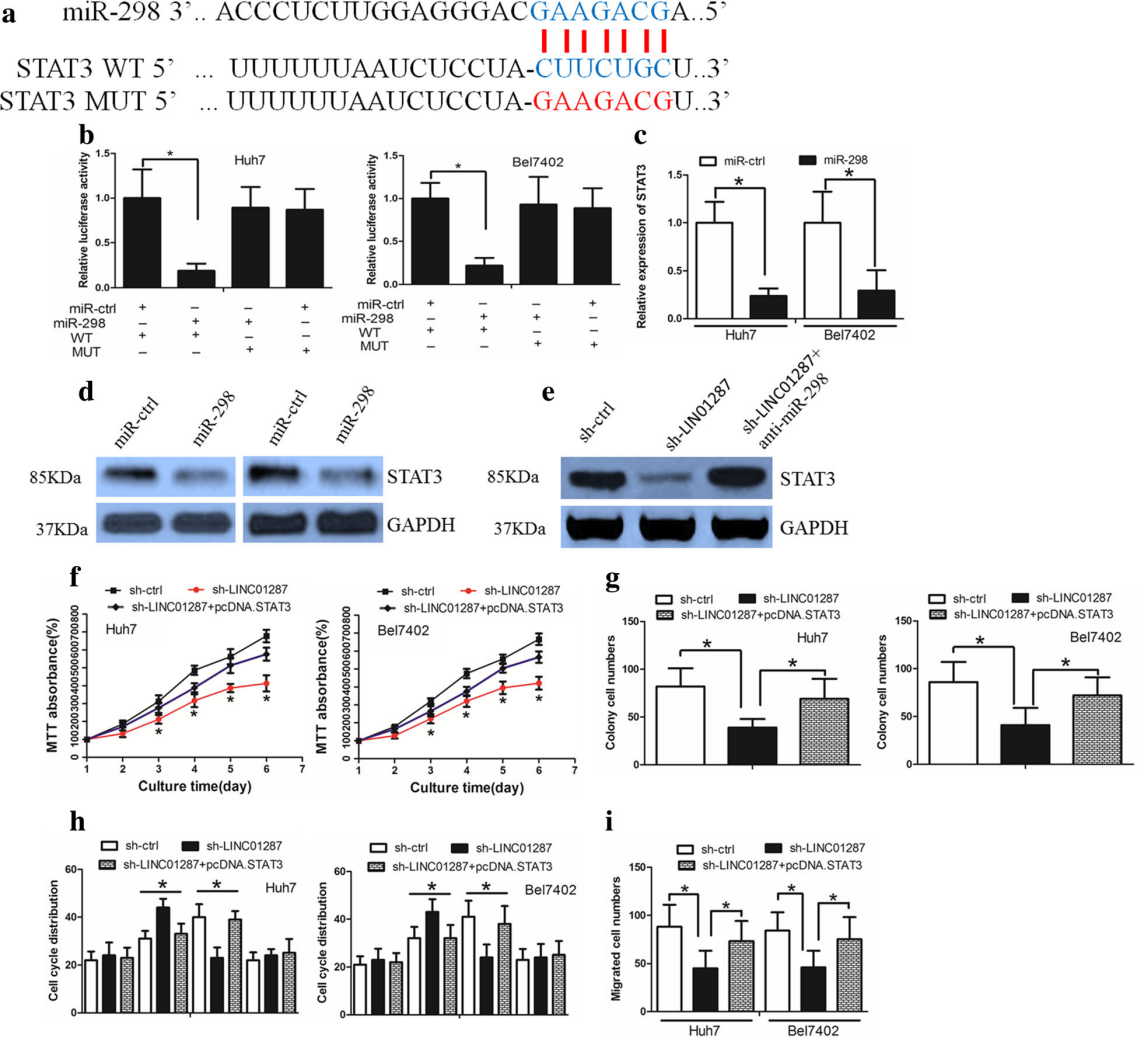
STAT3 was a down-stream target of miR-298. **a** The binding sites of miR-298 on STAT3. **b** The luciferase assay showed that cells transfected with miR-298 had less luciferase activity than those transfected with miR-ctrl. **c** miR-298 repressed STAT3 mRNA expression in HCC cells. **d** miR-298 repressed STAT3 protein expression in HCC cells. **e** Anti-miR-298 treatment led to the restoration of STAT3 expression in sh-LINC01287 cells. **f** The MTT assay revealed that sh-LINC01287 cells grew more slowly than sh-ctrl cells, while the overexpression of STAT3 rescued this effect. **g** The colony formation assay showed that sh-LINC01287 cells formed smaller and fewer colonies than the sh-ctrl cells, which was counteracted by the overexpression of STAT3. **h** LINC01287 down-regulation affected the cell cycle distribution, which was counteracted by the overexpression of STAT3. **i** LINC01287 down-regulation inhibited the invasion ability of HCC cells, which was rescued by the overexpression of STAT3

With the use of the software Targetscan, we identified a list of genes that may be down-stream targets of miR-298. STAT3 is a key master regulator that controls cancer proliferation and invasion, and thus we selected this protein for further study. The 3’-UTR region of STAT3 mRNA, including the predicted miR-298 recognition site (wild type) or the mutated sequence (mutant type), were subcloned into luciferase reporter plasmids (Fig. 4a). It was observed that miR-298 decreased luciferase activity in the wild type vector instead of that in the mutant type (Fig. 4b). Moreover, miR-298 decreased the expression of both STAT3 mRNA and protein (Fig. 4c and d). Thus, our data revealed that STAT3 is a potential target of miR-298.

Subsequently, we wanted to know whether LINC01287 exerts its function through the miR-298/STAT3 axis. We observed that LINC01287 inhibition decreased STAT3 expression. However, when miR-298 was inhibited, the effect of LINC01287 down-regulation on STAT3 expression was abolished (Fig. 4e). The overexpression of STAT3 was able to counteract the effect of LINC01287 down-regulation on cell growth, cell cycle distribution and invasion (Fig. 4f-i, Additional file 2: Figure S2D-F). We also knocked down miR-298 expression in sh-LINC01287 cells. We revealed that knockdown of miR-298 could counteract LINC01287 down-regulation’s effect on HCC cells (Additional file 2: Figure S2G-J).

Taken together, these data suggested that LINC01287 could exert its function through the miR-298/STAT3 axis.

### STAT3 elevated LINC01287 expression via c-Jun

**Fig. 5:**
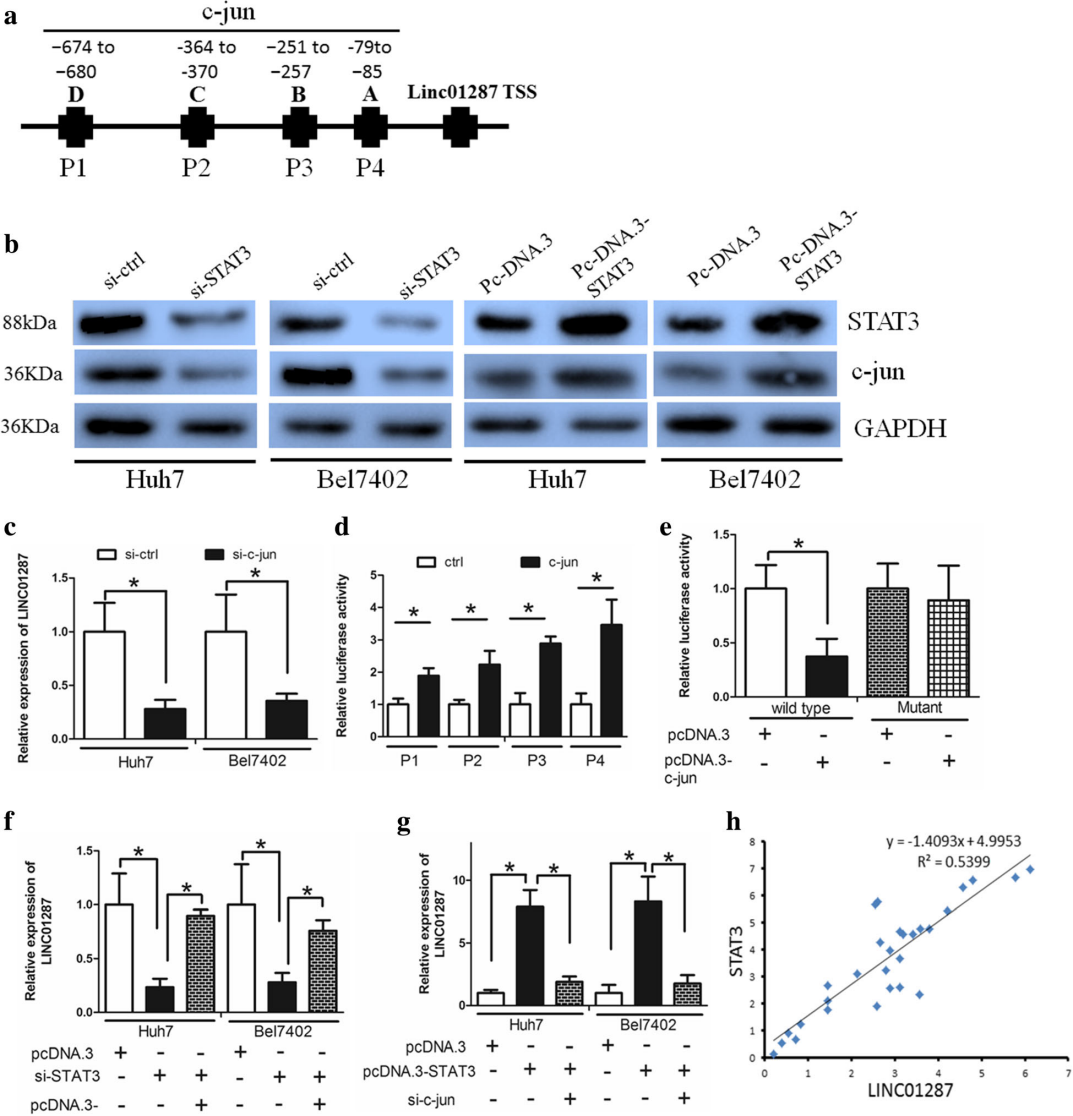
STAT3 elevated LINC01287 expression via c-jun. **a** The putative transcription factor-binding sites of c-jun in the LINC01287 promoter region. **b** Western blot revealed that STAT3 positively regulated c-jun expression. **c** Knockdown of c-jun decreased the expression of miR-298 in both Huh7 and Bel7402 cells. **d** An up-regulation of wild-type miR-298 promoter luciferase activity was observed upon up-regulation of c-jun in Huh cells. **e** When c-jun binding sites on LINC01287 were mutated, the effect of c-jun on LINC01287 in Huh7 cells was abolished. **f** In si-STAT3 cells, the forced overexpression of c-jun increased LINC01287 expression. **g** In HCC cells in which STAT3 was overexpressed, the knockdown of c-jun decreased LINC01287 expression. **h** LINC01287 expression was positively correlated with STAT3 expression in HCC

We next explored the existence of a feedback loop between STAT3 and LINC01287. We used the UCSC and PROMO bioinformatics software programs to analyze an 800-bp region upstream of the transcriptional start site (TSS) of LINC01287. Four c-jun-binding motifs at − 79 to − 85, − 251 to − 257, − 364 to − 370 and − 674 to − 680 were identified inside the putative promoter region upstream of the LINC01287 TSS. We named these transcription factor-binding sites (TFBSs) A, B, C and D (Fig. 5a). A previous study revealed that STAT3 expression elevated c-jun expression [20], and we assumed that STAT3 may regulate LINC01287 via c-jun. We revealed that c-jun expression was down-regulated in si-STAT3 cells. However, forced overexpression of STAT3 elevated c-jun expression (Fig. 5b). These data suggested that STAT3 positively regulates c-jun expression.

To explore whether c-jun increased LINC01287 expression, c-jun was down-regulated by specific siRNAs. The knockdown of c-jun decreased the expression of LINC01287 in both Huh7 and Bel7402 cells (Fig. 5c). To further confirm that c-jun binds to the LINC01287 promoter, chromatin immunoprecipitation and q-PCR assays were performed. An up-regulation of wild-type LINC01287 promoter luciferase activity was observed upon up-regulation of c-jun in Huh7 cells (Fig. 5d). Interestingly, when c-jun binding sites on LINC01287 were mutated, we observed that the effect of c-jun on LINC01287 was abolished (Fig. 5e). Considering all these findings, these data suggested that c-jun binds to the LINC01287 promoter and increases its expression.

In si-STAT3 cells, forced overexpression of c-jun increased the LINC01287 expression level (Fig. 5f). However, when STAT3 was overexpressed, the knockdown of c-jun decreased LINC01287 expression (Fig. 5g). Taken together, these findings suggested that STAT3 regulates LINC01287 expression via c-jun.

We further revealed that LINC01287 expression was positively correlated with STAT3 expression (Fig. 5h, Spearman’s correlation, *R* = 0.5399).

### Knockdown of lncRNA LINC01287 inhibited tumor growth in vivo

**Fig. 6:**
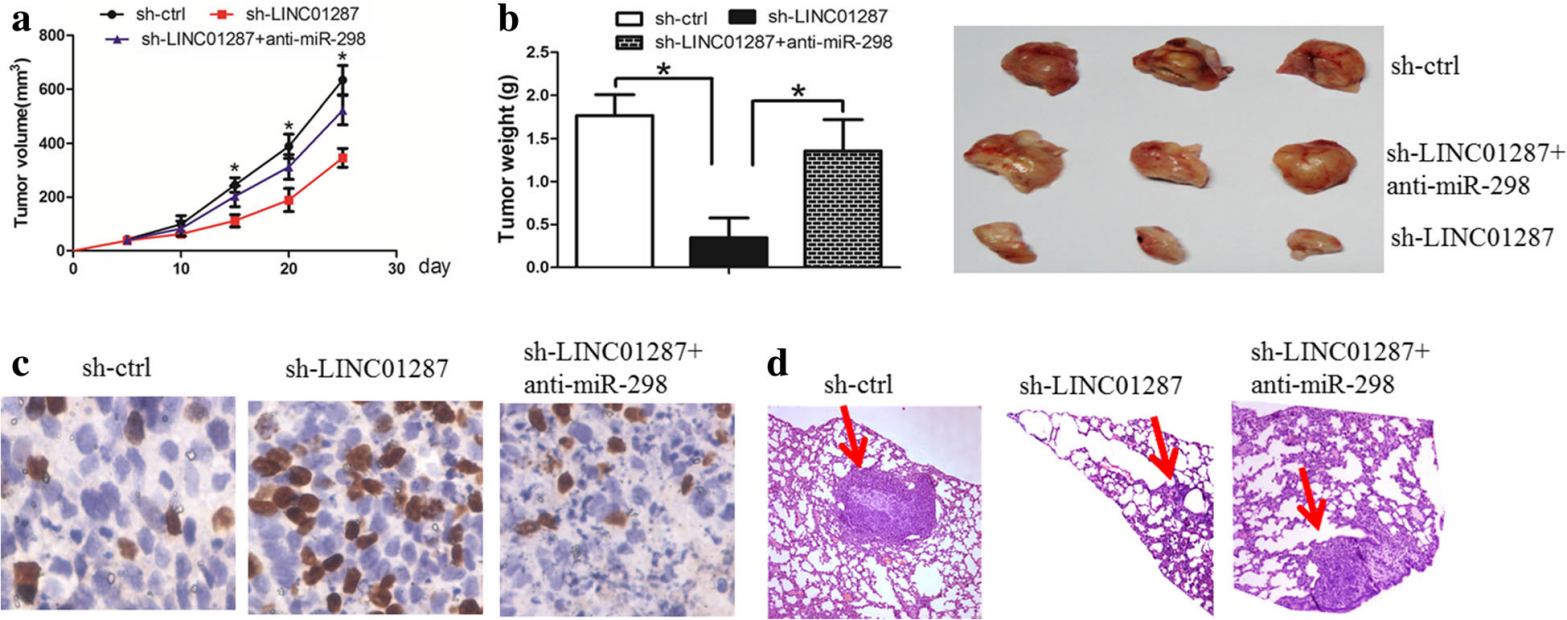
LINC01287 inhibition decreased tumor growth and invasion in vivo. **a** Compared with sh-ctrl cell-derived xenograft tumors, sh- LINC01287 cell-derived xenograft tumors grew more slowly, while anti-miR-298 treatment partly abolished this effect. **b** The mean weight of sh-LINC01287 cell-derived xenograft tumors was significantly lower than that of sh-ctrl cell-derived xenograft tumors, while anti-miR-298 treatment partly abolished this effect. **c** The knockdown of LINC01287 significantly decreased the percentage of Ki-67-positive cells in the tumors compared with the negative control group, while anti-miR-298 treatment partly abolished this effect. **d** The knockdown of LINC01287 decreased lung metastasis in vivo, while anti-miR-298 treatment partly abolished this effect

We then asked whether LINC01287 down-regulation inhibited tumor growth in vivo. When LINC01287 was inhibited, HCC cell-derived tumors grew more slowly. However, when miR-298 was inhibited in sh-LINC01287 cells, the LINC01287 down-regulation effect was partly abolished (Fig. 6a). The mean weight of the xenograft tumors was lower in the sh-LINC01287 group than in the sh-ctrl group and this effect was partly abolished by anti-miR-298 treatment (Fig. 6b). Tumor sections were stained for Ki-67 expression to quantitatively assess the proliferation index in the xenograft tumors; the proliferation index was lower in the sh-LINC01287 group and this effect was partly abolished by anti-miR-298 treatment (Fig. 6c). In addition, the knockdown of LINC01287 decreased the ability of these tumors to metastasize to the lungs in vivo and this effect was partly abolished by anti-miR-298 treatment (Fig. 6d). Taken together, these data supported the growth-promoting effect of LINC01287 and suggested that LINC01287 may exerted its function via miR-298 in vivo.

## Discussion

LncRNAs regulate multiple biological processes, including cancer cell growth and invasion [21]. Recent studies have suggested that lncRNAs can serve as effective therapeutic targets for cancer treatment. Recently, a list of lncRNAs have been reported to be implicated in regulating HCC proliferation, migration and invasion [22–24]. These literatures suggested that lncRNAs may be a useful strategy for HCC treatment.

In the present study, we used the TGCA database to explore lncRNAs that may be involved in HCC progression. LINC01287 was identified as an oncogene whose expression is significantly increased in HCC tissues. Furthermore, we revealed that LINC01287 was up-regulated in HCC cell lines and tissues. The biological role of LINC01287 has not been investigated before. We then performed functional study to explore the role of LINC01287 in HCC cells. The knockdown of LINC01287 inhibited cell proliferation and colony formation in vitro. The in vivo study revealed that LINC01287 down-regulation decreased tumor growth. In terms of its mechanism, LINC01287 down-regulation contributed to cell cycle arrest in G1 stage. The G1/S phase checkpoint proteins (e.g., c-myc, cyclin D1 and CDK4) were altered when LINC01287 was inhibited. These data suggested that LINC01287 may lead to cell growth via changes in cell cycle progression. We also revealed that LINC01287 down-regulation inhibited GC cell invasion in vitro and decreased lung metastasis in vivo. EMT plays a vital role in promoting cancer cell invasion [25]. We thus asked whether LINC01287 is involved in the EMT phenotype. It was revealed that LINC01287 down-regulation increased the expression of the epithelial marker E-cadherin, while it decreased the expression of the mesenchymal markers N-cadherin and Vimentin. These data suggested that LINC01287 may promote an EMT phenotype and thus lead to HCC cell invasion.

A previous study revealed that lncRNAs may act as endogenous molecular sponges that compete for miRNAs and negatively regulate miRNA expression [26, 27]. The interaction between lncRNAs and microRNAs have well been documented in cancer. For instance, lncRNA-PAGBC competitively binds to the tumour suppressive microRNAs miR-133b and miR-511 [28]. LncRNA FAL1 promotes cell proliferation and migration by acting as a ceRNA of miR-1236 in HCC cells [29]. These literatures prompted us to ask whether there was interaction between LINC01287 and miRNAs. Using online software, we identified several miRNAs that may interact with LINC01287. Among these miRNAs, we selected miR-298 for further study, since the expression level of miR-298 was significantly increased in sh-LINC01287 cells. Previous documents revealed that miR-298 was implicated in regulating cancer progression. MiR-298 was frequently down-regulated in cancer tissues and acted as a tumor suppressor by inhibiting cell proliferation and migration [30–32]. The role of miR-298 has seldom been reported in HCC, but we revealed that miR-298 may be a tumor suppressor in HCC. We also confirmed the regulatory relationship between LINC01287 and miR-298 based on the following data: 1) LINC01287 down-regulation increased miR-298 expression; 2) the luciferase activity assay confirmed the direct binding ability of the predicted miR-298 binding site on LINC01287; 3) The RIP assays found that LINC01287 and miR-298 were in the same RISC.

Emerging evidence has demonstrated the role of STAT3 in cancer progression, invasion and metastasis [33]. STAT3 was identified as a down-stream target of miR-298 in our study, and our data revealed that LINC01287 inhibition decreased STAT3 expression. However, when miR-298 was inhibited, the effect of LINC01287 down-regulation on STAT3 expression was abolished. The overexpression of STAT3 can counteract the effect of LINC01287 on HCC cells. These data suggested that LINC01287 exerts its function through the miR-298/STAT3 axis.

We further sought to determine the presence of a positive feedback loop between STAT3 and LINC01287. The recruitment of specific transcription factors often leads to abnormal lncRNA expression [34]. The transcription factor c-jun is a key regulator of cell growth [35] and metastasis [36] in cancer. We revealed four putative binding sites of c-jun in the region upstream of the LINC01287 locus. A subsequent experiment demonstrated that c-jun could positively regulate LINC01287 expression by directly binding to its promoter. Our study showed that STAT3 increased c-jun expression and therefore regulated LINC01287 expression. We further confirmed a positive correlation between STAT3 and LINC01287 in HCC tissues. Taken together, our findings revealed a feedback loop within the LINC01287/miR-298/STAT3 axis.

Overall, our data provided the first evidence that the LINC01287/miR-298/STAT3 axis controls cell growth and invasiveness of HCC cells. Therapeutics that target LINC01287 may therefore improve the treatment of HCC.

## Conclusions

In conclusion, our study demonstrated that LINC01287 was upregulated in HCC and may function as a ceRNA to increase STAT3 expression by sponging miR-298, which consequently contributes to HCC growth and metastasis. Our findings indicated that LINC01287 may be a potential therapeutic target for HCC treatment.

## Additional files

Additional file 1: **Figure S1.** (A) The TGCA database revealed that LINC01287 was up-regulated in HCC tissues. (B) The in vitro translation assay revealed that LINC01287 did not have coding ability. (C) LINC01287 was primarily expressed in the cytoplasm, as determined by RT-PCR. (TIF 4712 kb)

Additional file 2: **Figure S2.** (A) The colony formation assay demonstrated that oncogenic survival was significantly decreased in sh-LINC01287 cells compared with sh-ctrl cells. (B) sh-LINC01287 cells were significantly more likely to be in G1 phase and were less likely to be in S phase. (C) LINC01287 down-regulation decreased the invasion ability of HCC cells, as revealed by the Boyden assay. (D) The colony formation assay showed that sh-LINC01287 cells formed smaller and fewer colonies than the sh-ctrl cells, which was counteracted by the overexpression of STAT3. (E) LINC01287 down-regulation affected the cell cycle distribution, which was counteracted by the overexpression of STAT3. (F) LINC01287 down-regulation inhibited the invasion ability of HCC cells, which was rescued by the overexpression of STAT3. (G) The MTT assay revealed that LINC01287 down-regulation significantly decreased cell proliferation and the effect was counteracted by anti-miR-298 treatment. (H) The colony formation assay showed that sh-LINC01287 cells formed smaller and fewer colonies than the sh-ctrl cells, which was counteracted by anti-miR-298 treatment. (I) LINC01287 down-regulation affected the cell cycle distribution, which was counteracted by anti-miR-298 treatment. (J) LINC01287 down-regulation inhibited the invasion ability of HCC cells, which was rescued by anti-miR-298 treatment. (K) The putative microRNAs that may be regulated by LINC01287. (L) The expression levels of miR-298, miR-4308 and miR-23c were increased in sh-LINC01287 cells. (TIF 18965 kb)
