# Supplementary figures and images for: LINC01287/miR-298/STAT3 feedback loop regulates growth and the epithelial-to-mesenchymal transition phenotype in hepatocellular carcinoma cells
Source: J Exp Clin Cancer Res. 2018 Jul 13;37:149. doi: 10.1186/s13046-018-0831-2 (PMC6044102; doi:10.1186/s13046-018-0831-2)

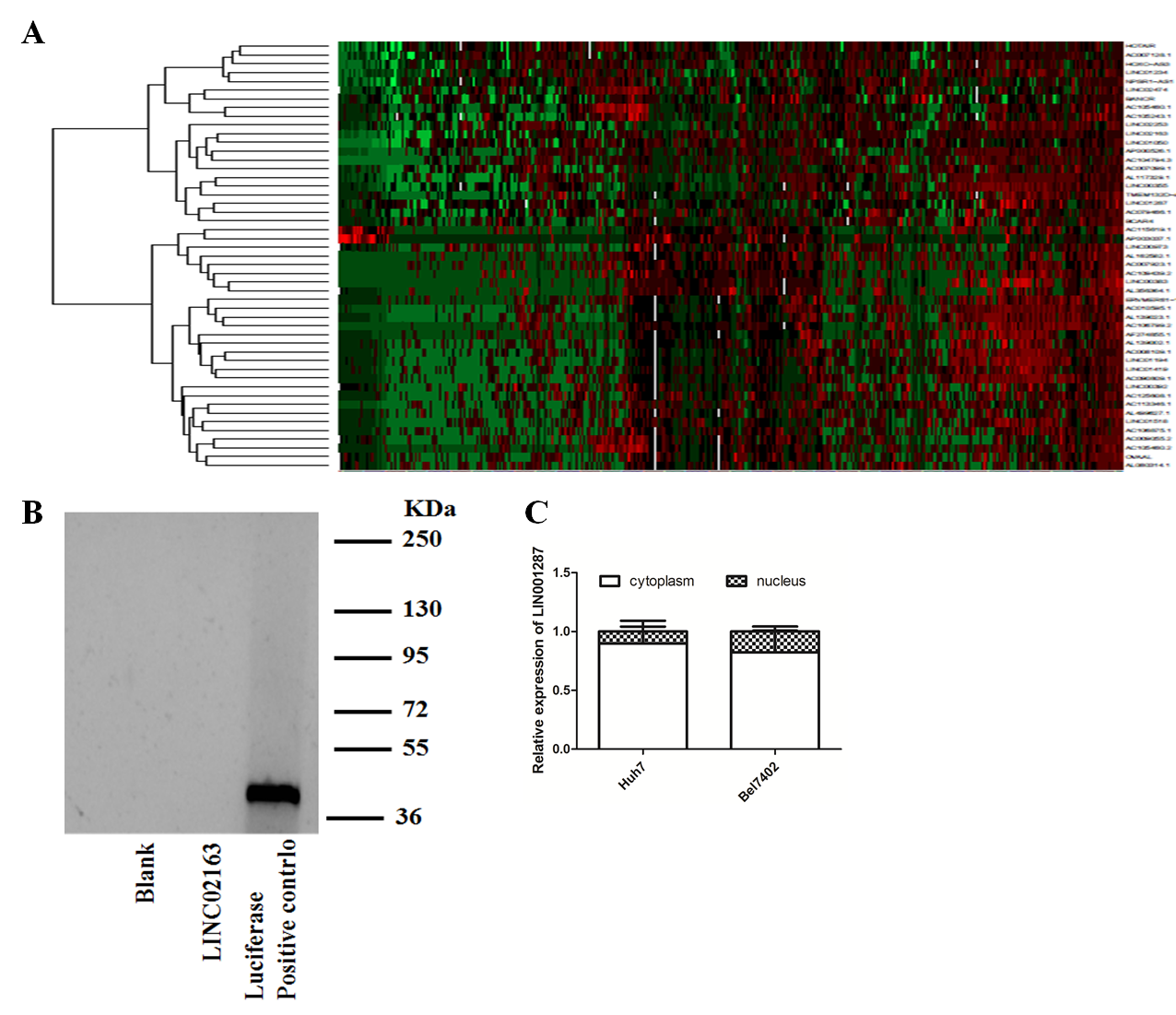

Supplement: Supplementary file 1 — Figure S1. (A) The TGCA database revealed that LINC01287 was up-regulated in HCC tissues. (B) The in vitro translation assay revealed that LINC01287 did not have coding ability. (C) LINC01287 was primarily expressed in the cytoplasm, as determined by RT-PCR. (TIF 4712 kb) [file 13046_2018_831_MOESM1_ESM.tif]

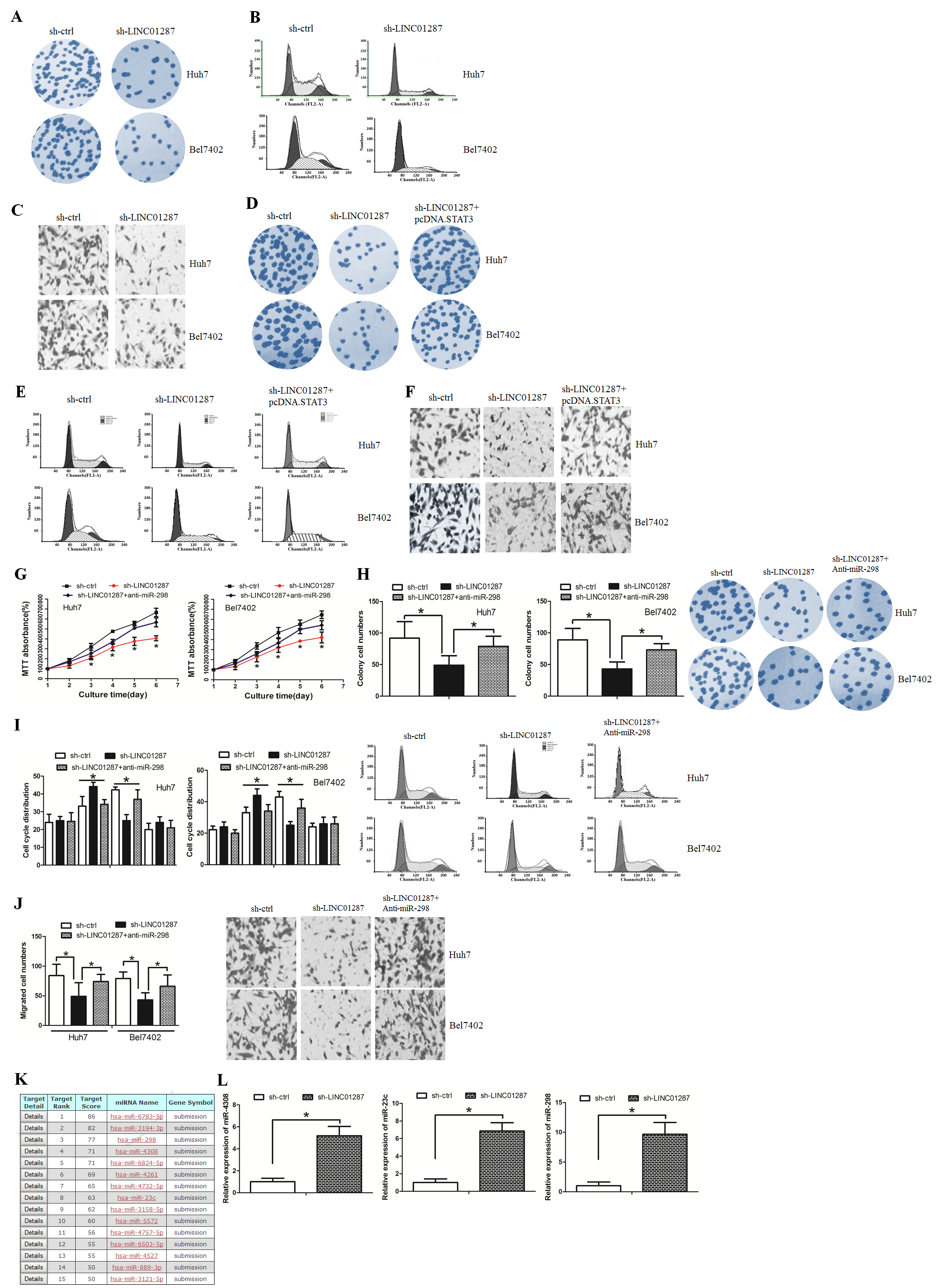

Supplement: Supplementary file 2 — Figure S2. (A) The colony formation assay demonstrated that oncogenic survival was significantly decreased in sh-LINC01287 cells compared with sh-ctrl cells. (B) sh-LINC01287 cells were significantly more likely to be in G1 phase and were less likely to be in S phase. (C) LINC01287 down-regulation decreased the invasion ability of HCC cells, as revealed by the Boyden assay. (D) The colony formation assay showed that sh-LINC01287 cells formed smaller and fewer colonies than the sh-ctrl cells, which was counteracted by the overexpression of STAT3. (E) LINC01287 down-regulation affected the cell cycle distribution, which was counteracted by the overexpression of STAT3. (F) LINC01287 down-regulation inhibited the invasion ability of HCC cells, which was rescued by the overexpression of STAT3. (G) The MTT assay revealed that LINC01287 down-regulation significantly decreased cell proliferation and the effect was counteracted by anti-miR-298 treatment. (H) The colony formation assay showed that sh-LINC01287 cells formed smaller and fewer colonies than the sh-ctrl cells, which was counteracted by anti-miR-298 treatment. (I) LINC01287 down-regulation affected the cell cycle distribution, which was counteracted by anti-miR-298 treatment. (J) LINC01287 down-regulation inhibited the invasion ability of HCC cells, which was rescued by anti-miR-298 treatment. (K) The putative microRNAs that may be regulated by LINC01287. (L) The expression levels of miR-298, miR-4308 and miR-23c were increased in sh-LINC01287 cells. (TIF 18965 kb) [file 13046_2018_831_MOESM2_ESM.tif]
